# Emotional responses and coping strategies in nurses and nursing students during Covid-19 outbreak: A comparative study

**DOI:** 10.1371/journal.pone.0237303

**Published:** 2020-08-07

**Authors:** Long Huang, Wansheng Lei, Fuming Xu, Hairong Liu, Liang Yu

**Affiliations:** 1 School of Psychology, Jiangxi Normal University, Nanchang, People’s Republic of China; 2 School of Humanities and Management, Wannan Medical College, Wuhu, People’s Republic of China; 3 School of Educational Science, Nanning Normal University, Nanning, People’s Republic of China; National Institutes of Health, UNITED STATES

## Abstract

The coronavirus disease (COVID-19) outbreak in December has seen more than 76,000 cases in China, causing more than 3,000 medical staff infections. As the disease is highly contagious, can be fatal in severe cases, and there are no specific medicines, it poses a huge threat to the life and health of nurses, leading to a severe impact on their emotional responses and coping strategies. Therefore, this study will investigate nurses’ emotional responses and coping styles, and conduct a comparative study with nursing college students. This study was conducted through the online survey ‘questionnaire star’ from February 1^st^ to February 20^th^, 2020 in Anhui Province, using the snowball sampling method to invite subjects. The results found that women showed more severe anxiety and fear than men. Participants from cities exhibited these symptoms more than participants from rural areas, however rural participants experienced more sadness than urban participants. The nearer a COVID-19 zone is to the participants, the stronger the anxiety and anger. The COVID-19 outbreak has placed immense pressure on hospitals and those nurses at the frontline are more seriously affected. Hospitals should focus on providing psychological support to nurses and training in coping strategies.

## Introduction

In December 2019, multiple unexplained cases of pneumonia were reported in Wuhan, Hubei Province, China. Epidemiological findings revealed severe human-to-human transmission, which was later confirmed to be caused by a novel corona virus (2019-nCoV) infection. The World Health Organization (WHO) named it Corona Virus Disease 2019 (COVID-19) [1]. The outbreak resulted in 13 prefecture-level cities in Hubei, including Wuhan, and one autonomous prefecture to announce closures from January 23, 2020. The Chinese government also announced the extension of the spring festival holidays (from seven days to 14 days), and implemented epidemic control measures such as suspension of schools, total shutdown and closure of businesses to reduce gathering of people and limit the spread of the virus. However, due to the large-scale population migration just before the Chinese Lunar New Year holidays, more than five million residents had reportedly left Wuhan to various other places in China and around the world. Compared to past outbreaks such as SARS and Ebola, COVID-19 is highly infectious during the incubation period, and asymptomatic infection persists. It can be transmitted through respiratory droplets, contact and aerosols [2]. As a result, COVID-19 has caused large-scale infections worldwide, leading to a pandemic. As of February 22, 2020, China has reported 51,699 confirmed cases (including Hong Kong, Macao, and Taiwan; this number includes 10,968 severe cases), a total of 22,888 discharged patients, 2442 deaths, and 76,936 confirmed cases. There were 4148 suspected cases. A total of 628,517 close contacts were tracked, and 106,089 close contacts were still in medical observation. A total of 1,719 confirmed cases and 17 deaths have occurred in other countries as of February 2020.

Due to the rapid spread of COVID-19, its strong contagion factor, lethality in severe cases, and no specific medication, it poses a huge threat to human life and health. The disease also has a huge impact on mental health, causing people to experience various degrees of emotional problems [3]. Seventeen years ago, the emergence of SARS in China also caused widespread fear and heightened emotions [4,5]. Therefore, we can predict that the outbreak of COVID-19 will cause considerable mass hysteria such as tension, anxiety, and fear which may lead to severe psychological disorders such as acute stress disorder, post-traumatic stress disorder, depression and suicide. Academician Zhong Nanshan, who leads the Chinese Health Commission, pointed out that psychological fear is more dreadful than the disease itself [6]. Although infectious diseases elicit a wide range of emotional responses, not everyone experiences the same degree of emotional impact [7]. Hospital medical staff is always at the forefront of any particular epidemic and they risk their lives to perform their duties. As they are more likely to be in close contact with COVID-19 patients, they are particularly vulnerable to infection and spreading the virus among colleagues and family members. To date, more than 3,000 medical staff have been infected with COVID-19, with six deaths amongst them. Such high number of infected medical personnel is unique in modern history. In addition to physical stress, medical staff also face huge mental burdens. This is particularly evident from the SARS and Ebola virus outbreaks [8,9]. Previous studies have found that SARS causes great pain to medical staff, and it causes much more pain to nurses than doctors [10]. This is due to the nature of their job, where it is important to be in close contact with infectious patients for long durations. In addition, many temporary hospital workers resigned during the outbreak, and a large part of their work had to be taken over by nurses. Therefore, the emotional problems of nurses during the COVID-19 epidemic deserve more attention.

COVID-19 has not only had an impact on people’s emotions, but their coping strategies too have undergone a change. Coping is defined as the thoughts and actions that individuals use to deal with stressful events [11]. Research has identified two general coping strategies: one is problem-focused coping where the purpose is to solve the problem or take action to change the status quo; and the other is emotion-focused coping, which aims to reduce the emotional distress associated with stressful situations [12]. Studies have found that emotions lead to specific coping strategies [13], and vice versa. Emotions are believed to have properties that motivate certain behaviors [13,14]. For example, fear is related to the desire to evade and protect themselves from incidents, anger leads to a desire to attack, disgust leads to a desire to expel, and happiness leads to a desire to entertain [13,15]. Moreover, emotions have been linked to the use of specific coping strategies [16]. In particular, adults who report more anger and fear prefer to use active-oriented coping strategies such as asking questions, while those who are sad are more likely to use non-active coping strategies such as avoiding or accepting problems [16]. In turn, the successful use of coping strategies will help individuals manage stressful events [11] and reduce negative emotions [12]. However, the direction of the relationship between emotional responses and coping strategies is not clear, and the relationship is not always constant. Some studies during the SARS epidemic have found that the relationship between them is age-specific [17]. So, the relationship between nurses’ coping strategies and emotional responses during a major infectious disease such as COVID-19 needs further research to clarify. To our knowledge, there has been no systematic assessment of the effects of COVID-19 on nurses’ emotional responses and coping strategies. Based on this, the purpose of this study was to explore the current status and relationship of emotional responses and coping strategies of nurses at all levels of hospitals in Anhui Province during the COVID-19 outbreak, and to compare them with non-front-line prospective nurses (nursing students).

## Methods

### Subjects

This study was conducted through an online survey with the questionnaire star, beginning on February 1, 2020 and ending on February 9, 2020 in Anhui Province, and the survey was approved by the ethical review board of Wannan Medical College. The snowball sampling method was used to invite subjects. An initial set of invitees (10 participants) was chosen to ensure a broad representation of age, gender, class of hospital, identity, and city. This group of invitees forwarded the questionnaire to ten of their colleagues or friends who they deemed suitable for the survey, and the second group forwarded the questionnaire in the same way. Participants were college students and first-line nurses aged 18–26 years old. They volunteered to participate in this study and signed an online informed consent form before collecting data. Respondents were excluded if they reported a history of mental illness and / or could not complete the online survey independently.

### Research tools

#### Demographic information

It mainly includes the basic information of the participants, such as gender, age, identity, rural or urban, whether there is a confirmed or isolated person in the community or administrative village (in order to evaluate the spatial distance of active COVID-19 cases from the participants).

#### Emotional responses

Referring to the positive and negative emotion (PANAS) scale, we design a negative emotional response scale to measure the public ‘s emotional response to COVID-19, including anxious, fear, sadness and anger, using a 5-point scale, ranging from 1 (no such emotion) to 5 (the most intense feeling of the emotion) (S1 File). The order of presentation for these items was randomized. Participants completed these quantitative projects and answered an open-ended question about their emotional response to the outbreak of COVID-19. Subsequently, two independent coders coded the answers, and the content of the code was the specific emotions expressed (reliability between raters from 0.80 to 0.91). The scores of these items were cross validated through qualitative measurement of emotional responses. The modes of qualitative and quantitative measurement results are almost the same. With this in mind, we conducted the following analysis based on the scores of quantitative indicators. The Cronbach’s α coefficients of scale is 0.77 in this study.

#### Coping strategies

The tool for measuring the coping strategies during the outbreak of COVID-19 was revised based on the Brief COPE prepared by Carver (1997) [17,18]. Yeung and Fung (2013) used it to measure residents’ response strategies during the SARS outbreak, and it displayed a good reliability. The scale consists of problem-focused coping (active coping, planning, and use of instrumental support) and emotion-focused coping (use of emotional support, acceptance, positive reframing, religion, humor, substance use, self-distraction, self-blame, denial, behavior disengagement, and venting) consisting of 2 subscales with a total of 16 entries. We asked participants to report how often they used the strategy described in each project to respond to COVID-19, ranging from 1 (none) to 5 (always) on 5 levels. Higher scores indicated higher levels of coping. In this study, the Cronbach’s α coefficients of problem-focused and emotion-focused coping categories are 0.817 and 0.811 respectively.

#### Statistical analyses

Data was analyzed using SPSS 21 software for data statistics. The t-test or one-way analysis of variance was used to test the differences in emotional responses and coping styles among categorical variables. Pearson linear correlation analysis was used to test the correlation between emotional responses and coping styles. Multiple linear regression analysis was used to determine the interaction between emotional responses and coping styles. All regression analysis with independent observations (Durbin-Watson) does not exhibit multicollinearity and significant outliers (There is no studentized deletion of observations with residuals greater than 3 times the standard deviation, data leverage values are less than 0.2, and there is no value of Cook’s distance greater than 1); *p* <0.05 was considered statistically significant.

## Results

### General information

A total of 850 questionnaires were sent, and 802 valid and complete questionnaires were recovered, with a recovery rate of 94.35%. The participants’ ages ranged from 18 to 84 years old (mean 21.31±2.71 years); 602 (74.9%) were female, 202 (25.1%) were male; 298 (37.1%) were in rural areas and 506 (62.9%) were in urban areas; 374 participants (46.5%) were nurses (mean 23.95±1.37 years) and 430 participants (53.5%) were nursing students (mean 19.00±0.84 years); 189 (50.5%) were in Class III, 185 (49.5%) were in Class II; 377 (46.9%) were from cities with severe epidemics (Hefei, Fuyang, Bengbu, where the number of confirmed diagnoses is > 100), 170 (21.1%) were from cities with moderate epidemic levels (Anqing, Wuhu, Lu’an, Suzhou, Ma’anshan with confirmed diagnosis between 30–100), 257 (32.0%) were from prefecture-level cities (Xuancheng, Huaibei, Huainan, Chizhou, Quzhou and Huangshan with the number of confirmed diagnoses < 30). Sixty-one participants (7.6%) were active in communities with confirmed patients, 168 (20.9%) were active in communities with isolated people, and 575 (71.5%) participants lived in communities not affected by the epidemic (Table 1).

**Table 1:** Sample description.

	Number of cases	Composition ratio (%)
**Identity**		
Nurse	374	46.5
Nursing college students	430	53.5
**Class of hospital**		
Class III	180	50.5
Class II	194	49.5
**Gender**		
Male	202	25.1
Female	602	74.9
**Rural or urban**		
Urban	506	62.9
Rural	298	37.1
**Severity of the city**		
Light	257	32.0
Medium	170	21.1
Serious	377	46.9
**Spatial distance**		
Far	575	71.5
Medium	168	20.9
Near	61	7.6

### Descriptive statistics of emotion responses

Independent sample t test found that anxiety in professional nurses (t_(799.33)_ = 3.05, *p* = 0.002, Cohen’s d = 0.220), fear (t_(799.33)_ = 3.05, *p* <0.0001, Cohen’s d = 0.326), sadness (t_(799.33)_ = 4.59, *p* <0.0001, Cohen’s d = 0.565), and anger (t_(802)_ = 4.56, *p* <0.0001, Cohen’s d = 0.322) was significantly higher than the emotional level of nursing college students. Women experienced significantly higher anxiety than men (t_(802)_ = -3.62, *p* <0.0001, Cohen’s d = 0.08) and fear (t_(314.44)_ = 5.17, *p* <0.0001, Cohen’s d = 0.427). Participants from rural areas experienced more sadness (t_(584.85)_ = -3.85, *p* <0.0001, Cohen’s d = 0.285) than participants from urban areas, while participants from urban areas experienced more anxiety (t_(679.52)_ = 2.55, *p* = 0.009, Cohen’s d = 0.192) and anger (t_(802)_ = 3.04, *p* = 0.002, Cohen’s d = 0.226) than participants who lived in rural areas. Details are provided in Table 2.

**Table 2:** Descriptive statistics of emotion responses.

		Anxiety				Fear				Sadness				Anger			
		M(SD)	*p*	*d / η*_*p*_^*2*^	*power*	M(SD)	*p*	*d / η*_*p*_^*2*^	*power*	M(SD)	*p*	*d / η*_*p*_^*2*^	*power*	M(SD)	*p*	*d / η*_*p*_^*2*^	*power*
**Identity**	**Nurse**	3.00(0.94)	0.002	0.220	0.929	3.16(0.92)	<0.0001	0.326	0.999	2.84(0.90)	<0.0001	0.565	1.000	2.20(0.90)	0.000	0.322	0.998
	**Nursing college students**	2.77(1.14)				2.82(1.15)				2.26(1.14)				1.90(0.96)			
**Class of hospital**	**Class III**	3.06(0.98)	0.249	0.128	0.339	3.11(0.94)	0.409	0.086	0.209	2.83(0.96)	0.901	0.021	0.075	2.16(0.93)	0.380	0.089	0.215
	**Class II**	2.94(0.90)				3.19(0.91)				2.85(0.90)				2.24(0.87)			
**Sex**	**Male**	2.64(0.99)	<0.0001	0.301	0.980	2.63(1.14)	<0.0001	0.427	1.000	2.47(1.09)	0.348	0.074	0.230	2.06(0.98)	0.708	0.031	0.104
	**Female**	2.95(1.07)				3.09(1.01)				2.55(1.08)				2.03(0.93)			
**Rural or urban**	**Urban**	2.95(1.10)	0.009	0.192	0.837	2.94(1.05)	0.253	0.084	0.311	2.41(1.04)	<0.0001	0.285	0.988	2.11(0.95)	0.002	0.226	0.926
	**Rural**	2.75(0.98)				3.03(1.09)				2.72(1.13)				1.90(0.91)			
**Severity of the city**	**Light**	2.79(1.01)	0.226	0.004	0.318	2.94(0.96)	0.422	0.002	0.318	2.52(1.05)	0.636	0.001	0.124	2.08(0.90)	0.051	0.008	0.618
	**Medium**	2.95(1.09)				3.07(1.15)				2.47(1.15)				2.16(1.08)			
	**Serious**	2.90(1.08)				2.96(1.10)				2.56(1.09)				1.95(0.90)			
**Spatial distance**	**Far**	2.78(1.05)	<0.0001	0.023	0.977	3.03(1.10)	0.073	0.007	0.523	2.49(1.12)	0.246	0.003	0.302	1.92(0.89)	<0.0001	0.045	1.000
	**Medium**	3.03(0.91)				2.88(0.96)				2.63(0.98)				2.25(0.96)			
	**Near**	3.31(1.34)				2.77(096)				2.64(0.97)				2.56(1.09)			

One-way ANOVAs found that anxiety, fear, sadness and anger had no significant differences in relation to the severity of urban outbreaks. There were differences in anxiety levels in relation to spatial distance of COVID-19 cases (F_(2,677)_ = 9.26, *p* <0.0001, η_p_^2^ = 0.023), multiple comparisons found that anxiety in near (*p* <0.0001) and medium (*p* = 0.008) space distance are significantly greater than when covid-19 cases are further away. Anger emotions have significant differences in the spatial distance of COVID-19 (F_(2,801)_ = 18.78, *p* <0.0001, η_p_^2^ = 0.045). Multiple comparisons found that the anger emotions in different spatial distances from large to small are—near, medium, and far (*ps* <0.0001) (Table 2).

#### Descriptive statistics of coping strategies

The independent sample t test found that more number of nurses used problem-focused coping methods than nursing students (t_(802)_ = 4.99, *p* <0.0001). Results showed significantly more women than men took to problem-focused coping (t_(317)_ = -2.30, *p* = 0.022). More men than women took to emotion-focused coping (t_(264.75)_ = 4.47, *p* <0.0001). Nurses in Class II hospitals are more emotionally coping than nurses in Class III hospitals (t_(338.74)_ = 2.539, *p* = 0.018) (Table 3).

**Table 3:** Descriptive statistics of coping strategies.

		Problem-focused coping				Emotion-focused coping			
		M(SD)	*p*	*d / ηp2*	*power*	M(SD)	*p*	*d / ηp2*	*power*
**Identity**	**Nurse**	2.66(0.63)	<0.0001	0.355	0.999	1.80(0.42)	0.165	0.11	0.462
	**Nursing college students**	2.42(0.72)				1.75(0.49)			
**Hospital level**	**Class III**	2.72(0.58)	0.121	0.176	0.519	1.74(0.31)	0.018	0.268	0.827
	**Class II**	2.61(0.67)				1.85(0.49)			
**Sex**	**Male**	2.43(0.75)	0.022	0.197	0.781	1.92(0.59)	<0.0001	0.397	0.999
	**Female**	2.57(0.67)				1.72(0.40)			
**Rural or urban**	**Urban**	2.56(0.68)	0.129	0.101	0.399	1.76(0.45)	0.243	0.086	0.32
	**Rural**	2.49(0.70)				1.80(0.48)			
**Severity of the city**	**Light**	2.51(0.71)	0.717	0.001	0.103	1.81(0.53)	0.107	0.006	0.457
	**Medium**	2.54(0.72)				1.71(0.49)			
	**Serious**	2.55(0.67)				1.78(0.39)			
**Spatial distance**	**Far**	2.53(0.72)	0.446	0.002	0.189	1.72(0.41)	<0.0001	0.033	0.998
	**Medium**	2.59(0.63)				1.93(0.56)			
	**Near**	2.47(0.59)				1.75(0.49)			

One-way ANOVAs found that three levels of severity did not differ in their use of problem-focused coping and emotion-focused coping toward COVID-19. Problem-focused coping had no significant difference at different spatial distances; whereas emotion-focused coping had significant differences at different spatial distances (F_(2,801)_ = 13.55, *p*<0.0001, η_p_^2^ = 0.033). Multiple comparisons found that the emotion-focused coping at medium space distances were significantly higher than near space distances (*p* = 0.007) and far space distance (*p* <0.0001) (Table 3).

#### Correlation between emotional responses and coping strategies

After controlling for gender, spatial distance, identity, and urban-rural attributes, the Pearson linear correlation analysis was performed on the emotional response and coping strategies of both groups. Pearson linear correlation analysis showed that in addition to sadness, other variables correlated with each other (e.g., anger and problem-focused coping) (*ps* <0.001) (Table 4).

**Table 4:** Correlations among emotion responses and coping strategies.

	**Anxiety**	**Fear**	**Sadness**	**Anger**	**Problem-focused coping**	**Emotion-focused coping**
**Anxiety**	1					
**Fear**	0.60***	1				
**Sadness**	0.46***	0.39***	1			
**Anger**	0.48***	0.36***	0.49***	1		
**Problem-focused coping**	0.17***	0.21***	0.07	0.13***	1	
**Emotion-focused coping**	0.10**	0.11**	0.06	0.12***	0.61***	1

* *p*<0.05,

** *p*<0.01,

*** *p*<0.001

#### Regression analysis of emotional responses and coping strategies

Multiple regression analysis was carried out to reveal coping strategies’ impact on emotion response. Only problem-focused coping proved to be the influencing factor for nurses’ anxiety (R^2^ = 0.045, R^2^ = 0.044, *β* = 0.190, *p* = 0.001), as indicated by the model at 4.4%. Problem-focused coping (*β* = 0.396, *p* <0.0001) and emotion-focused coping (*β* = -0.151, *p* = 0.06) were the influence factors for nurses’ fear emotions(R^2^ = 0.124, adjusted R^2^ = 0.119), indicated by the model at 11.9%. Emotion-focused coping were the influence factors for nurses’ anger emotions (R^2^ = 0.028, adjusted R^2^ = 0.023, *β* = 0.117, *p* = 0.043), indicated by the model at 2.3%. For nursing students, only problem-focused coping influenced their anxiety (R^2^ = 0.023, adjusted R^2^ = 0.019, *β* = 0.184, *p* = 0.004) and fear (R^2^ = 0.023, R^2^ = 0.018, *β* = 0.165, *p* = 0.009), indicated by the model at 1.9% and 1.8% respectively (Table 5).

**Table 5:** Results of multiple linear regression analysis of emotion responses.

Variables	Anxiety				Fear				Sadness				Anger			
	*β*	*t*	*P*	95%CI	*β*	*t*	*p*	95%CI	*β*	*t*	*p*	95%CI	*β*	*t*	*p*	95%CI
**Nurse**																
**Constant**	2.025	8.417	<0.0001	1.552–2.498	2.207	9.699	<0.0001	1.760–2.655	2.581	10.642	<0.0001	2.104–3.058	1.455	6.274	<0.0001	0.999–1.911
**Problem-focused coping**	0.190	3.318	0.001	0.115–0.451	0.396	7.205	<0.0001	0.423–0.741	-0.025	-0.419	0.675	-0.205–0.133	0.077	1.336	0.182	-0.052–0.272
**Emotion-focused coping**	0.054	0.948	0.344	-0.131–0.374	-0.151	-2.751	0.006	-0.572–0.095	0.089	1.526	0.128	-0.057–0.452	0.117	2.027	0.043	-0.007–0.494
**Nursing students**																
**Constant**	2.304	10.698	<0.0001	1.881–2.727	2.280	10.520	<0.0001	1.854–2.706	1.878	8.687	0.000	1.453–2.303	1.369	7.550	<0.0001	1.012–1.725
**Problem-focused coping**	0.184	2.921	0.004	0.095–0.486	0.165	2.626	0.009	0.066–0.459	0.057	0.896	0.371	-0.107–0.286	0.043	0.683	0.495	-0.107–0.222
**Emotion-focused coping**	-0.059	-0.931	0.352	-0.423–0.151	-0.023	-0.373	0.709	-0.344–0.234	0.040	0.627	0.531	-0.196–0.380	0.114	1.816	0.070	-0.018–0.465

Multiple regression analysis was conducted to reveal emotion responses’ impact on coping strategies. Only fear emotions influenced nurses’ problem-focused coping score (R^2^ = 0.119, adjusted R^2^ = 0.109, *β* = 0.305, *p* <0.0001), indicated by the model at 10.9%. Furthermore, nursing students’ emotion-focused coping score was influenced by the anger emotion (R^2^ = 0.022, adjusted R^2^ = 0.013, *β* = 0.143, *p* = 0.017), as indicated by the model at 1.3% (Table 6).

**Table 6:** Results of multiple linear regression analysis of coping strategies.

Independent variables	Dependent variable							
	Problem-focused coping				Emotion-focused coping			
	*β*	*t*	*p*	95%CI	*β*	*t*	*p*	95%CI
**Nurse**								
**Constant**	1.993	14.711	<0.0001	1.727–2.260	1.597	16.861	<0.0001	1.411–1.783
**Anxiety**	0.075	1.056	0.292	-0.044–0.145	0.096	1.280	0.201	-0.023–0.109
**Fear**	0.305	5.322	<0.0001	0.131–0.284	-0.046	-0.760	0.448	-0.074–0.033
**Anger**	-0.102	-1.916	0.056	-0.140–0.002	0.027	0.484	0.628	-0.037–0.062
**Sadness**	0.038	0.610	0.542	-0.060–0.114	0.098	1.493	0.136	-0.015–0.107
**Nursing students**								
**Constant**	2.069	19.566	<0.0001	1.861–2.277	1.588	21.960	<0.0001	1.446–1.730
**Anxiety**	0.076	1.198	0.232	-0.031–0.126	-0.021	-0.335	0.738	-0.063–0.045
**Fear**	0.096	1.550	0.122	-0.016–0.136	0.057	0.924	0.356	-0.028–0.077
**Anger**	-0.040	-0.632	0.528	-0.103–0.053	-0.021	-0.336	0.737	-0.063–0.044
**Sadness**	0.077	1.302	0.194	-0.030–0.146	0.143	2.407	0.017	0.013–0.133

## Discussions

COVID-19 has been a source of great stress for both individuals and social groups. Different individuals experience different levels of psychological crisis, but it is especially harder for those at the core of the crisis. The study found that nurses at the frontline exhibit stronger anxiety, fear, sadness, and anger than nursing students. Although, when facing an epidemic, nursing students also undergo extreme psychological stress and get concerned about their career ahead. They also experience a range of feelings such as excitement, doubt, and helplessness [19]. However, nurses face far more psychological stress than nursing students due to their working environment. Concerns about being infected during close contact with patients, unfamiliarity with new specialized working environments and procedures, physical discomfort caused by special protection, witnessing patient suffering and death, and long-term separation from family members—all these factors cause extreme psychological stress to medical staff. The inability to be physically present with their family due to infection concerns further adds to the nurses’ distress. They also undergo a sense of defeat at witnessing a declining patient despite all their medical interventions, leading to feelings of guilt and self-blame. When a patient complains, nurses feel aggrieved and not understood. Due to a shortage in medical staff, nurses are faced with added physical, mental and environmental stimuli, which leads to increased psychological load and more serious emotional problems [20]. This result is consistent with the results of a recent study by Xu and Zhang [21], who found that 85.37% (35 patients) of the first-line nurses fighting COVID-19 had emotional reactions, including two nurses with depression, 16 nurses with anxiety, and 21 nurses with terror. During the SARS outbreak, many nurses had conflicting roles as medical service providers and parents. On the one hand, they felt altruistic and professionally responsible. On the other hand, they were afraid and guilty that they might infect their families [22]. Nickell (2004)’s study found that about 20% of the population suffered from depression during SARS, and the incidence of nurses is as high as 45% in Toronto [23].

Long-term research has revealed that women suffer significantly higher levels of depression, anxiety, and loneliness than men. This is considered to be related to gender traits. Women themselves attach more importance to their inner experiences and self-perceptions, their emotions are more fragile and sensitive, and they are more vulnerable to depression, anxiety and loneliness [24]. Our study supports this thought. During the SARS outbreak, Gao et al. (2003) found that more women than men seek counseling for emotional issues [24]. This further indicates that there are gender differences in psychological reactions in the face of a public health emergency. This study also found that participants from urban areas expressed more anxiety and fear than participants from rural areas. However, rural participants experienced more sadness than urban participants. This may be due to the fact that cities are densely populated and has a large concentration of people. The COVID-19 disease is more evident in participants from urban areas than rural areas, raising their concerns about catching the infection and leading to more anxiety and fear. On the contrary, rural participants pay more attention to others who maybe ill and experience sadness for them. In addition, we found that the severity of the epidemic in cities do not make a huge difference to individual emotions, which may indicate that individuals are not very concerned about the severity of the epidemic in the city. This point could also be due to the small difference in the severity of the epidemic in the cities selected in our study. However, this study found that spatial distance between the epidemic and participants notably affected individual emotions. It was also revealed that participants who lived in the same community or administrative village that was affected by COVID-19 (with diagnosed patients or isolators) had stronger anxiety and anger than those who lived in unaffected zones. The presence of diagnosed patients in a community means a higher probability of being infected. The geographical location of COVID-19 patients and the spatial distance of participants are a reflection of psychological distance. The closer the psychological distance is, the more people feel the danger, the more sexual and threatening, the more intense anxiety and anger. Our results and the research results during the outbreak of SARS support this conclusion [25].

College students often develop immature or negative coping strategies instead of positive problem solving methods when faced with pressure caused by public health emergencies [26]. As a medical worker with clinical work experience, the nurse has more knowledge in facing the same crisis. This is consistent with our research, which found that nurses are more proactive in using problem-focused coping than nursing students. This study also found that women are more likely to opt for problem-focused coping mechanisms than men, and less likely to pick emotion-focused coping strategies. As mentioned earlier, women are more vulnerable and sensitive to emotions, therefore emotion-focused coping is rarely used when dealing with stress. In addition, this study also found that participants in affected zones were more likely to cope with emotion-focused strategies than those in unaffected communities. This is because being in an unaffected area, the participants do not focus much on COVID-19 and do not cause a strong emotional response to the pandemic. When a member of the community does test positive (i.e., when the spatial distance closes in), participants may not experience sufficient emotion-focused coping due to the psychological typhoon eye effect [27], which means that closer to the event center is less affected.

In a subsequent regression analysis, we found that the problem-coping style is the common influencing factor for anxiety in nurses and nursing students. This indicates that the more measures they take to deal with the epidemic, the more the anxiety. However, due to the low interpretability of the model, they are at 4.4% and 1.9%, respectively. In the future, it is necessary to carry out longitudinal follow-up research to explore whether the adoption of problem-coping styles does lead to an increase in anxiety. In addition, we found that problem coping styles and emotional-coping styles are influencing factors of nurses’ fear emotions, and here the model’s explanatory power reaches 11.9%. The only factor that affects the nursing students’ fear emotions is the problem-coping style, explained by the model at 1.8%, indicating that the fear of nursing students is less affected by problem-coping styles. This may be because nursing students generally have a low level of fear and therefore are not easily affected by coping styles. The fear of nurses is more closely related to coping styles, which may be because nurses have more resources and opportunities to understand COVID-19 and its implications. Higher awareness leads to better grasping of the highly contagious and lethal nature of this new virus, resulting in more fear and the phenomenon "the more you cope, the more you fear”. In the subsequent regression analysis of the impact of emotional responses on coping mechanisms, we found that fear is the influencing factor for nurses’ problem-coping styles, and the explanatory power is 10.9%. This means that the more apprehensive the nurse is, the more he/she will seek to deal with the problem. This phenomenon was not found in nursing students. This may be because the nurses’ fear factor is much higher than students’, and the nurse is also more capable of adopting corresponding coping strategies to cope with their tensions.

## Conclusion

During the COVID-19 outbreak, factors such as gender, urban/rural, and spatial distance influenced anxiety, fear, sadness, anger and coping strategies of nurses and nursing students. Nurses have stronger emotional responses and are more willing to adopt problem-focused coping than nursing students. This study further explores the relationship between emotional responses and coping strategies. There may be a cycle of "more coping-more panic" among nurses, not necessarily among nursing students. In the aftermath of COVID-19, we suggest hospitals focus on the following steps–provide more psychological support to nurses, adopt better training in coping strategies, arrange for adequate medical protective equipment, and develop a broad range of interventions to block the spread of infectious diseases so as to form a safe environment where COVID-19 stops spreading in hospitals. This will create an optimistic environment and guarantee the personal safety of nurses, thereby enabling them to carry on with the highest quality of patient care to win the battle against this epidemic.

## Supporting information

S1 File Emotional responses scale. (DOC)
