# Supplementary material for: Emotional responses and coping strategies in nurses and nursing students during Covid-19 outbreak: A comparative study
Source: PLoS One. 2020 Aug 7;15(8):e0237303. doi: 10.1371/journal.pone.0237303 (PMC7413410; doi:10.1371/journal.pone.0237303)
Supplement: S1 File — (DOC) [file pone.0237303.s001.doc]

**S1 File 1 : Emotional** **responses scale**

Guidance: Please answer according to your own true feelings in the past two weeks during the COVID-19. 1= "Not at all", 2= "A little", 3= "Fair", 4= "Strong", 5= "Very strong".

| Scale | 1= "Not at all", 2= "A little", 3= "Fair", 4= "Strong", 5= "Very strong" |
| --- | --- |
| 1 | Anxious |
| 2 | Fear |
| 3 | Sadness |
| 4 | Anger |
